# Mouse models as a tool to study asymptomatic DENV infections

**DOI:** 10.3389/fcimb.2025.1554090

**Published:** 2025-03-26

**Authors:** Paulo Henriques, Alexandra Rosa, Helena Caldeira-Araújo, Ana Margarida Vigário

**Affiliations:** ^1^ Projecto Medicina, Faculdade de Ciências da Vida, Universidade da Madeira, Funchal, Portugal; ^2^ Centro de Química da Madeira (CQM), Universidade da Madeira, Funchal, Portugal; ^3^ Gulbenkian Institute for Molecular Medicine, Lisbon, Portugal

**Keywords:** dengue, mouse models, asymptomatic infections, inapparent infections, dengue virus, dengue fever, immune response

## Abstract

Asymptomatic outcome accounts for most dengue virus infections and is likely to play an important role in maintaining virus circulation, contributing to its dissemination and shortening inter-epidemic periods. While dengue immunopathogenesis, investigation of potential therapeutics, and vaccine efficacy have been widely studied, only recently have inapparent infections begun to be comprehensively addressed as an integral and important part of the puzzle that is dengue infection. Animal models are one of the tools utilized to study dengue and, among these, mouse models have played an important role in understanding both dengue pathogenesis and the hosts’ initial immune response. However, these models have mostly focused on untangling the drivers of disease severity ignoring asymptomatic dengue virus infections. In this mini-review, the authors propose to provide a concise overview of the current state-of-the-art of existing mouse models with potential use for studying asymptomatic dengue virus infections, elaborating on the pros and cons of the several models. Variations in experimental conditions, such as altering the viral load of the inoculum or employing different virus entry routes, especially in mice with partial or transient blockade of the type I interferon response, might be sufficient to obtain both symptomatic and asymptomatic viremic mice. This would enable the study of factors involved in asymptomatic dengue virus infections.

## Introduction

Dengue virus (DENV) is a mosquito-borne flavivirus responsible for dengue, an emerging infectious disease that has become a major health concern in tropical and subtropical regions (reviewed in Halstead (2007); Guzman et al. (2010). Caused by any of its four circulating serotypes (DENV1 to DENV4), dengue usually manifests as either an undifferentiated febrile episode or classical Dengue Fever (DF) that can progress to more severe and potentially lethal Dengue Hemorrhagic Fever or Shock-Syndrome. Several signs and symptoms such as fever, headache, nausea, vomiting, myalgia, arthralgia, rash, and leukopenia characterize DF, whilst, in the more severe cases, hemorrhage, plasma leakage, breathing difficulty, and organ dysfunction also occur. While much attention has been focused on symptomatic infections, most DENV infections are asymptomatic (Balmaseda et al., 2010; Johansson et al., 2014), as with many other flaviviruses. Asymptomatic DENV infections refer to infections with detectable viremia, but without any of the clinical symptoms described above, that are usually associated with DF. These may also include infections with very mild or non-specific symptoms that cannot be clearly attributed to DENV infection, sometimes globally referred to as inapparent dengue infections.

Understanding DENV infection and its outcomes remains challenging due to the complex nature of the virus, its intricate interactions with the host immune system (reviewed in (Guzman et al., 2010; Henriques et al., 2023), and the limitations of many of the existing mouse models.

DF is primarily associated with humans, where DENV is thought to evade the immune response by targeting key components of the type I Interferon (IFN) response. For instance, several DENV non-structural proteins have been described to disrupt type I IFN signaling in humans by degrading STAT2 (Jones et al., 2005; Mazzon et al., 2009; Ashour et al., 2010), inhibiting STAT1 phosphorylation (Muñoz-Jordán et al., 2003, 2005) or cleaving STING (Aguirre et al., 2012). Type I IFN signaling is critical for controlling viral replication during the early stages of infection, and its inhibition allows efficient viral replication in human cells. In contrast, DENV seems unable to evade the rodent immune response through the inhibition of type I IFN signaling (Yauch and Shresta, 2008), leading to their inherent resistance to DENV infection. As a result, dengue research has been hindered by the difficulty of establishing mouse models that reproducibly allow DENV infection and competently mimic the disease as it manifests in humans. Nevertheless, several immunocompetent, immunocompromised, and even humanized mouse models have been employed using different strategies and experimental designs to characterize specific features of DF pathogenesis, as well as, evaluate potential therapeutics and vaccine candidates, with varying degrees of success (reviewed in Chen and Diamond (2020) and Coronel-Ruiz et al. (2020)). Yet, asymptomatic DENV infections are the most common outcome in humans and are likely central to understanding the host factors influencing the clinical outcome. However, to our knowledge, asymptomatic DENV infections have been seldomly comprehensively addressed using mouse models, likely due to their limitations in achieving DENV infection and effective viremia without disease signs or animal mortality.

The authors hereby propose to briefly revisit the existing mouse models for studying DF pathogenesis, and to further objectively discuss the feasibility of using these models to study asymptomatic DENV infections.

## Key assumptions and challenges in studying asymptomatic DENV infection using mouse models

Animal models rarely fully replicate the primary manifestations observed in human clinical infections. Nevertheless, in our understanding, if certain key assumptions can be met, we may be able to use mouse models to successfully investigate viral and host factors contributing to an asymptomatic outcome of DENV infection. Firstly, the appropriate mouse models must allow DENV infection to occur, ideally in all inoculated animals, with the least possible manipulation of their immune system. Secondly, the infection should be self-resolved within a few days without any manifestations/signs in its course. These models should then be compared with models in which, under similar experimental conditions, all animals develop viremia and DF signs. Alternatively, models could be set up where all the animals develop viremia, but only a proportion exhibit DF-associated signs. This would allow for comparative studies between animals with and without disease signs within the same model and experimental settings.

These theoretical assumptions lead us, however, to two important challenges to overcome. On the one hand, mouse models where all inoculated animals become infected need to be established while, at the same time, criteria and protocols must be defined to accurately confirm the establishment of infection. For instance, in some studies, animals did not have detectable infectious particles in their serum, as assessed by either RT-PCR or plaque assays, probably because the levels were below the test sensitivity, but viral RNA or infectious particles were present in various tissues (Shresta et al., 2004; Paes et al., 2005; Sridharan et al., 2013; Wilken et al., 2023) and/or seroconversion was identified (Shresta et al., 2004; Tan et al., 2010). On the other hand, it is crucial to define which behavioral signs and laboratory findings should be considered as indicative of DF, and which may be associated with asymptomatic infection. Mice with DF have hematological and biochemical alterations, such as leukopenia with lymphopenia, thrombocytopenia, and elevated aminotransferases and gamma-glutamyl transpeptidase. Asymptomatic or inapparent DENV infections are also expected to induce, at a certain extent, changes in at least some of these parameters, because of the immune response to the virus, and in some cases mild unspecific illness manifestations. In addition, the development and validation of a tool that allows the assessment of observable parameters would help to define the physiological state of the mice. For instance, the adaptation of SHIRPA test (**S**mithKline Beecham, **H**arwell, **I**mperial College, **R**oyal London Hospital, **p**henotype **a**ssessment), a battery of standardized tests used for qualitative behavioral and functional assessment in mouse studies to determine phenotypes based on scores, may be a useful tool to assess symptomatic DF in mice, and to a lesser extent, asymptomatic infections. Among the parameters to evaluate DF, we can suggest exploratory behavior (motor performance), muscular tone (limb strength), reflexes and self-preservation (touch escape and aggression), hygiene-related behavior (grooming) (Carroll et al., 2010) all as indicators of animal’s general health and well-being, as they may relate to myalgia, arthralgia, headache, extreme fatigue, and/or malaise known to occur in human symptomatic infections. In addition, loss of weight may relate to loss of appetite, and/or gastrointestinal signs such as nausea, vomiting or diarrhea (Wilken et al., 2023). Some of these signs may be expected to change slightly in asymptomatic infections. Given the inter-individual variability observed in all animal models, adopting a scoring system based on the sum of major/minor criteria could be a reliable approach for both behavioral signs and laboratory findings, that might include a cut-off score for asymptomatic infections.

## Lessons from existing models for studying asymptomatic DENV infections

### Immunocompetent mice

As mentioned above, immunocompetent mice such as A/J, C57BL/6 and BALB/c tend to be naturally resistant to DENV. However, their infection can be successfully achieved by manipulating experimental conditions, highly reliant on the used DENV strain (including mouse-adapted and non-mouse-adapted strains), the route of inoculation, and/or the size of the inoculum (Huang et al., 2000; Shresta et al., 2004; Paes et al., 2005; Chen et al., 2007; Gonçalves et al., 2012).

Although A/J mice seem to be more susceptible to DENV infection than other models (Huang et al., 2000), they may not be helpful for studying asymptomatic infections. Following DENV-2 intravenous inoculation, most animals develop paralysis, with detectable infectious particles in the central nervous system, increased hematocrit, leukopenia (Shresta et al., 2004), and transient thrombocytopenia (Huang et al., 2000), usually leading to death, even in the absence of signs. Neurological manifestations are uncommon in humans, and much less likely is that apparently asymptomatic infections could lead to death. Moreover, Shresta et al. (2004) reported that nearly half of A/J mice lacked disease signs, but these cannot be assumed to represent true asymptomatic as viremia was not tested in these animals.

In our opinion, the findings in C57BL/6 and BALB/c mice make them the more promising immunocompetent mice for establishing asymptomatic DENV infections. Intraperitoneal inoculation of a mouse-adapted DENV-1 caused viremia and detectable viral particles in the brain, kidney, and liver of C57BL/6 mice, along with thrombocytopenia, spleen hemorrhage, and liver damage, but no evident clinical signs (Gonçalves et al., 2012). Paes et al. (2005) reported that BALB/c mice, infected intraperitoneally with a non-mouse-adapted DENV-2 strain, developed liver pathology, as indicated by elevated ALT/AST levels and hepatic lesions, despite low viremia and the absence of other clinical symptoms.

Immunocompetent mice benefit from an intact immune system, and despite their limitations, we believe that an optimized combination of experimental conditions - such as the choice of DENV strain, inoculum size, and inoculation route - could aid in developing models to investigate certain mechanisms of asymptomatic DENV infections, as complementary evidence to findings in other models.

### Mice with deficiencies in innate immune response

To overcome the inherent resistance of immunocompetent mice and to study different aspects of the DENV infection, animals with deficiencies in innate host defense responses have been used. AG129 mice, which lack receptors for both IFN type I (IFN-α/β) and type II (IFN-γ) on a 129/Sv or 129/SvEv background, have shown to be more susceptible to DENV infection than wildtype (WT) mice, presenting vascular leakage, changes in blood cell counts (platelets, lymphocytes, and erythrocytes), and rapidly developing severe disseminated disease or progressing to death (Johnson and Roehrig, 1999; Milligan et al., 2017; Sarathy et al., 2018; Chen and Diamond, 2020). Consequently, these models are likely to have limited use in the study of asymptomatic DENV infections. Nevertheless, they demonstrated that inoculation route, dose and viral strain influence the severity and type of clinical signs, with DENV-1 (Baldon et al., 2022) or low-passage DENV3 isolate reported as non-lethal in AG129 mice (Sarathy et al., 2018). However, it remains unclear whether lowering the inoculum dosage and/or using those isolates could produce asymptomatic infections in these animals. Conversely, *Ifnar^-/-^* (A129) mice typically exhibit greater resistance to DENV than AG129 and result in lower mortality (Shresta et al., 2004; Prestwood et al., 2012). Given their ability to support DENV infection while retaining IFN-γ signaling, *Ifnar* ^-/-^ mice have been useful in probing mechanisms of DENV pathogenesis in a less immunocompromised model (Shresta et al., 2004; Chen and Diamond, 2020). Notwithstanding, DENV could be detected in peripheral organs, including the liver and spleen, as well as in blood (Tan et al., 2010; Sarathy et al., 2015). Early DENV viral load seems to be limited by both IFN-α/β and IFN-γ receptors pathways (Shresta et al., 2004), while IFN-γ receptor also plays a critical role in late central nervous system infection clearance (Prestwood et al., 2012). Indeed, A129 mice had lower viral RNA in spinal cords and brains than AG129 mice confirming that IFN signaling has a role in protecting the central nervous system from DENV infection (Prestwood et al., 2012). Considering that A129 mice did not present clinical symptoms when infected with DENV-2, even though infectious particles were found in multiple tissues, this may be an interesting model to complement the study of asymptomatic DENV infection.

Likewise, *STAT1* and *STAT2*-deficient mice have also contributed to unraveling the initial IFN-mediated response to DENV infection. Shresta et al. (2005) observed that the initial viral clearance is STAT1-pathway-dependent, while later viral clearance and protection against DF is partially due to a STAT1-independent IFN response, where STAT2 seems to be involved (Perry et al., 2011) and to promote NK and B cells activation and MHC class I upregulation on macrophage and dendritic cells’ surface. Also, inhibition of IFN-α and IFN-β production seems to be incomplete in these models, since detectable levels have been observed within a few hours post-infection (Perry et al., 2011).

In these models with an initial blockade of the type I IFN response, DENV inoculation resulted in detectable viremia, and allowed both symptomatic and asymptomatic outcomes in *Stat1* ^-/-^ mice (Shresta et al., 2005), while *Stat2* ^-/-^ mice were all asymptomatic (Perry et al., 2011). However, since most studies focus on specific disease phenotypes, such as death by paralysis, not all clinical signs representative of the full spectrum of the disease were screened for, and thus some may have been present but were not detected in *Stat2* ^-/-^ mice (Shresta et al., 2005; Perry et al., 2011). In the light of these results, these models are also likely suitable for the study of asymptomatic DENV infections, targeted to immune responses that might be hampered in other models that completely inhibit the initial IFN response.

Perry et al. (2009) found that both *Cardif* ^-/-^ and WT C57BL/6 mice were susceptible to infection when intravenously injected with different inoculum doses of DENV-2 serotype, with no apparent signs of disease or mortality, while *Ifnar* ^-/-^ mice showed 100% mortality at the higher inoculum. However, *Cardif* ^-/-^ displayed 10-fold higher levels of DENV RNA in serum, bone marrow, and lymphoid tissues within the first 18 hours of infection, when compared to WT mice, supporting the essential role of Cardif in triggering IFN response, and the observed delay in IFN production in its absence. As far as we known, the *Cardif* ^-/-^model was not further explored in the context of dengue, likely because it did not display apparent disease signs, and most studies focus on pathogenesis of symptomatic dengue and vaccination strategies. However, this model fulfills the main assumptions we consider necessary for studying asymptomatic DENV infection, allowing a complementary approach, with immune response blockade at Cardif level. Possible improvements could be achieved by changing the serotype/strain, route, or dose of the inoculum, to verify the feasibility that a proportion of animals develop dengue signs.

### Mice with transient suppression or conditional deletion of type I signaling

A transient suppression of the IFN-I signaling using MAR1-5A3, an anti-IFNAR1 monoclonal antibody, was shown to render WT mice more permissive to flavivirus replication and dissemination (Pinto et al., 2011; Lazear et al., 2016; Wilken et al., 2023), with limited influence on their adaptative immune response (Sheehan et al., 2006). Using this approach, Chuong et al. (2020) showed that C57BL/6J mice, pretreated with MAR1-5A3, and inoculated via a combined intradermal/subcutaneous route on the following day with low-passage isolates of DENV-1 or DENV-2, generated viremia and mild hematological changes, depending on the isolate, with no evident clinical signs being reported. Wilken et al. (2023) also demonstrated that C57BL/6J mice with MAR1-5A3 treatment prior to inoculation with a non-mouse-adapted DENV-2 strain exhibited no disease signs over 15 days, as measured by scores of weight loss, appearance, activity, and gastrointestinal symptoms. Animals sacrificed on day 5 showed splenomegaly, but no other gross pathological changes, and had detectable viral RNA in sera and visceral organs. In contrast, *Ifnar^-/-^* mice in this same study developed clinical signs of disease, such as rapid weight loss, and showed 1000-fold higher viral levels than WT MAR1-5A3-treated mice, 2-3 days post-infection (Wilken et al., 2023).

The strategy used in the above-mentioned studies seems to be a good basis for studying inapparent DENV infections, without genetic manipulation of the animals. An increasing dose of MAR1-5A3 or viral inoculum could potentially promote symptomatic infections in C57BL/6J mice, allowing the comparison of different study groups within the same model. Also, it would be interesting to compare with *Ifnar^-/-^* mice, under the same experimental conditions except for MAR1-5A3-treatment, since this immunocompromised model has a complete and permanent blockage at the same level of type I IFN signaling and could allow an insight over the entire spectrum of the infection, from asymptomatic to severe dengue.

Another possible approach is the conditional knockout of *Ifnar* in specific subsets of immune cells. This would render animals more permissive to infection but, at the same time, allowing type I IFN response in other cells to help in the resolution of the infection. Züst et al. (2014) showed that CD11c-Cre *Ifna* ^rfl/fl^ mice, which lack IFNAR expression primarily in dendritic cells, develop viremia with a slight loss of weight, but most mice recover completely when infected intraperitoneally with a non-mouse-adapted virus DENV-2. Although other signs of disease, such as hematological changes, were not assessed in this study, these could be suitable for studying asymptomatic infections.

### Humanized mouse models

Humanized mouse models have also significantly advanced our understanding about diverse aspects of dengue infection (reviewed in Yuya et al. (2024)). Importantly, they have been shown to sustain DENV infection, with detectable viraemia and clinical signs such as erythema, thrombocytopenia and fever (reviewed in Yuya et al. (2024)). Once again, these findings vary according to the infecting genotype (Mota and Rico-Hesse, 2009; Frias-Staheli et al., 2014) and route of infection: subcutaneous inoculation leads to higher infection rate and prolonged viremia compared to intraperitoneal inoculation (Jaiswal et al., 2009), while mosquito-bite inoculation results in higher and sustained viremia, and more severe disease (Cox et al., 2012). Additionally, most studies showed that viremia in humanized mice is achieved with lower-dose DENV inoculum than in other models. Given their engraftment with human immune cells, humanized mouse models are among the closest resembling human DENV infection. By combining a low viral load with an inoculation route resulting in reduced viremia and clinical signs, they may offer valuable insights into asymptomatic DENV infection. Moreover, they may be used to infer differences in susceptibility to infection and immune response, due to intrinsic characteristics of different human engraftment donors. However, these models are not without limitations and pose several challenges that need to be overcome, including significant variability in engraftment success, which can hinder the replication of results, as well as their higher cost, time and labor, compared to other models.

## Concluding remarks

Currently, no mouse model fully recapitulates the clinical spectra of human DENV infection. This is particularly relevant for the asymptomatic outcome, as studies have mainly focused on the most severe consequences and the protection associated with vaccines. For this reason, and since mice are inherently more resistant to infection by these viruses than humans, research has relied heavily on immunodeficient mice, which most often develop severe symptoms and/or die. However, immunocompetent mice together with transiently immunocompromised mice on type I IFN signaling, have the potential to become good models for studying asymptomatic infections, as long as inoculation routes, virus strains, and doses are adapted (**Table 1**). Indeed, some authors have already highlighted the potential of some of these models (Baldon et al., 2022; Wilken et al., 2023). As of this point, the main obstacle to establishing a universal model to study asymptomatic infections appears to be the greatly variable results, which mainly depend on the strain used and/or the number of *in vitro* passages. In addition, in order to be able to study asymptomatic infections using mouse models, establishing precise and standardized methods to detect infection and protocols to assess the signs of disease/infection that accurately distinguish dengue fever from asymptomatic infection in mice are also important challenges to overcome.

**Table 1:** **Sumary of the most relevant models to study dengue and advantages and limitations of their use to study asymptomatic DENV infection**.

	Mouse model	Background	DENV serotype	Inoculum	Entry route	Viremia (% mice)	Signs	References	Advantages and limitations of the potential use as asymptomatic model	
									Advantages	Limitations
Wild-type	A/J	A/J	DENV2	10^8^ PFU	IV	+ / ns	transient thrombocymiddleenia; paralysis	Huang et al., 2000	immunocompetent mice; different inoculum dosage, DENV serotype and entry route may allow asymptomatic infections	neurological commitment is not a common manifestation in human infections
			DENV2	10^8^ PFU	IV	0%	increased hematocrit; leukopenia; paralysis; death	Shresta et al., 2004		
	BALB/c	BALB/c	DENV2	10^4^ TCID_50_/0.2 ml	IP	75%	liver damage	Paes et al., 2005		
	C57BL/6	C57BL/6	DENV1	7.2 × 10^7^ PFU	IP	100%	liver damage; thrombocymiddleenia; spleen hemorrhage	Gonçalves et al., 2012		
	MAR1-5A3	C57BL/6	DENV1	*ns*	ID/SC	100%	mild hematological changes; no signs of disease	Chuong et al., 2020	immunocompetent mice; transient blockade of type 1 IFN response; may allow asymptomatic infections	
		C57BL/6	DENV2	*ns*	ID/SC	100%	mild hematological changes; no signs of disease			
		C57BL/6	DENV2	10^6^ PFU	SC	0%	no signs of disease; splenomegaly	Wilken et al., 2023		
Immunocompromised	AG129	129/SvEv	DENV2	10^6^ PFU	IP	+ / ns	paralysis; blindness; death	Johnson and Roehrig, 1999		no asymptomatic infections are observed; paralysis and/or death are not common manifestations in human infections
		129/Sv	DENV2	10^7^ - 10^2^ PFU	IP	+ / ns	ruffled fur; lethargy; diarrhoea-lyke symptoms; death	Tan et al., 2010		
		129/Sv	DENV4	10^7^ PFU	IP	100%	weight loss; limited motility; hunched posture; death	Sarathy et al., 2015		
		*ns*	DENV1	*ns*	IP	≥50%	vascular leakage; thrombocymiddleenia; leukopenia; increased hematocrit; death	Milligan et al., 2017		
		*ns*	DENV3	10^7.5^ FFU	IP	+ / ns	weight loss; leukopenia; thrombocymiddleenia	Sarathy et al., 2018		
		*ns*	DENV1	10^6^ PFU	IP	+ / ns	*ns*	Baldon et al., 2022		
	STAT1^-/-^	129/SvEv	DENV2	10^8^ PFU	IV	+ / ns	paralysis	Shresta et al., 2005	incomplete blockade of type 1 IFN response; allows asymptomatic infection	neurological commitment is not a common manifestation in human infections
			DENV1	4.4 × 10^4^ PFU		*nd*	paralysis			
	Cardif ^-/-^	C57BL/6	DENV2	3 × 10^6^ PFU	IV	+ / ns	no signs of disease	Perry et al., 2009		
	STAT2^-/-^	129/SvEv	DENV2	2 × 10^5^ PFU	IV	+ / ns	no signs of disease	Perry et al., 2011		
	A129	129/Sv	DENV2	3 × 10^6^ PFU	IV	+ / ns	no signs of disease	Prestwood et al., 2012		
Humanized	RAG2^-/-^γ_c_^-/-^	BALB/c	DENV2	10^6^ PFU	IP/SC	≥50%	fever	Kuruvilla et al., 2007	allows the study of human cells' response to DENV; may allow asymptomatic infection	low replicability due to grafting variability; high cost
	NOD-*scid IL2rγ^null^* (NSG)	NOD	DENV2	10^6^ PFU	IP/SC	0 - 100%	weight loss; ruffled fur; hunched back posture; rash	Jaiswal et al., 2009		
		NOD	DENV2	10^6^ PFU	SC	+ / ns	erythema; fever; thrombocymiddleenia	Mota and Rico-Hesse, 2009		
	NSG-BLT HLA-A2	NOD	DENV2	10^6^ PFU	SC	100%	weight loss; ruffled fur; hunched back posture	Jaiswal et al., 2012		
	NSG-BLT	NOD	DENV2	1 × 10^6^ TCID_50_	IV	20 - 40%	weight loss; ruffler fur; hunching; thrombocymiddleenia	Frias-Staheli et al., 2014		

ns, not specified; nd, not determined; IV, intravenous; ID, intradermal; IP, intraperitoneal; SC, subcutaneous; PFU, plaque forming unit; FFU, focus forming unit.
